# Digital design and virtual simulation of sports bras based on TG3D-Nuno knitted fabric simulation

**DOI:** 10.1371/journal.pone.0350668

**Published:** 2026-06-11

**Authors:** Liushan Tao, Anhua Zhong

**Affiliations:** Wuhan Textile University, School of Fashion, Wuhan, Hubei Province, People’s Republic of China; TU Dublin Blanchardstown Campus: Technological University Dublin - Blanchardstown Campus, IRELAND

## Abstract

In response to the key issues in the current digital design of sports underwear, such as fabric simulation distortion, disconnection between dynamic and static verification, and lagging functional evaluation, this study proposes a full-process digital development method for rib-knitted sports underwear based on the collaboration of TG3D-Nuno and CLO3D. Taking cotton/spandex (78%/22%) rib-knitted fabric as the research object, its multi-dimensional physical properties such as color, normal, roughness, and displacement are collected through the TG3D-Nuno 3D scanning system. After parameter mapping and calibration, the high-fidelity reproduction of the fabric’s visual texture and mechanical properties is achieved in the CLO3D virtual environment. Moreover, combined with a standard female human model (height 175.3 cm, bust 80.6 cm), the pattern of a functional sports underwear integrating a front-opening zipper, crossed shoulder strap structure, and elastic hem is designed using the “3D pen” tool. Furthermore, by constructing a dynamic simulation scenario in a running posture, a systematic verification of the underwear’s fit, support stability, and fabric collaborative deformation characteristics during exercise is carried out. The experimental results show that the digital process established in this study significantly improves the virtual restoration accuracy of knitted fabrics. Based on the evaluation of the size matching degree index, the 3D matching degree of the optimized pattern in key areas exceeds 98%. The dynamic simulation effectively identifies and corrects functional defects such as shoulder strap slippage and insufficient cup fit. This method realizes a complete technical closed-loop from fabric digitization to dynamic function verification, providing a feasible technical solution for the rapid development and precise design of sports underwear.

## 1. Introduction

The sports bra is an essential piece of equipment for women [1]. If the design of sports underwear fails to adequately meet the requirements of ergonomics and support performance, the resulting chest discomfort may significantly limit women’s participation in sports [2]. Although enhancing chest support can reduce breast displacement during physical activities, breast movement control and bra fit remain the primary breast-related concerns of women [3]. However, in the clothing industry, the pain points of the traditional model, such as “long R & D cycle, high trial-and-error cost, and insufficient personalized supply,” have become increasingly prominent. Virtual simulation technology, with its advantages of visualization, quantifiability, and iterability, has become the core supporting technology to solve these problems [4]. Among them, sports underwear, as a typical knitted product with both functionality and body-fitting properties, often encounters problems such as poor pattern body-fitting, high frequency of design repair, and long cycle during its design and production processes [5]. Therefore, by relying on the accurate digital expression of the physical properties and appearance texture of knitted fabrics, as well as the realistic simulation of the dynamic interaction process between the fabric and the human body, the long-standing problems in the design and production of sports underwear can be reduced. Compared with loose-fitting clothing, the digital research of body-fitting knitted products such as sports underwear faces more complex challenges [6]. On the one hand, the degree of simulation of knitted fabrics directly determines the visual simulation effect of underwear and the physical properties of the product. The elasticity of the fabric affects the pressure distribution of sports underwear, thus influencing the pressure comfort and support performance of sports underwear [7]. On the other hand, the core functions of sports underwear are strongly related to human movement postures, and it is necessary to accurately simulate the clothing deformation and support performance in dynamic scenarios such as running [8]. In addition, the complex curved surface structure of the female chest further increases the technical difficulty of the collaborative simulation of the pattern and the fabric [9].

At present, the digital simulation research of knitted underwear shows obvious branching characteristics. One category of research focuses on improving the simulation accuracy of knitted fabrics [10–13]. For example, Zhang Hui et al. achieved high-precision scanning of knitted fabric textures based on an improved machine vision algorithm. However, their results only remained at the level of texture digitization and did not extend to the dynamic simulation of clothing [14]. Another example is that scholars Chen Yushan et al. established the knitting pattern model, loop geometry, and grid model of the “double-tied-mouth” special structure of weft-knitted tubular seamless fabrics, and clarified the structural characteristics of the double-layer loop at the tied-mouth [15]. However, their simulation object focused on the fabric itself, without combining with a real human model to complete the dynamic simulation of the clothing wearing effect. Moreover, it was only for simple seamless bandeau products and did not involve the complex structures of functional underwear.

Another category focuses on the digital production of clothing based on human morphology [5,8,16–18]，However, the absence of a collaborative mechanism between these aspects is particularly acute, and it has become a crucial bottleneck restricting the digital research and development level of sports underwear. For instance, scholar Guo Mengke applied the UVW unwrapping function from the game-design field to the design of female knitted underwear prototypes. She obtained models through 3D body scanning, generated initial prototypes through UVW unwrapping and proportion calculation, and then optimized them using CLO3D software virtual fitting technology. Ultimately, she developed a dart-less and well-fitting knitted underwear prototype, thus innovating the clothing pattern-making method [18]. However, this research solely focuses on the digital generation and optimization of the underwear pattern. It doesn’t involve the digital simulation of the knitted fabric itself, nor does it relate to dynamic wearing scenarios. Additionally, scholars like Hong Wenjin proposed a design method for intelligent sports underwear based on Clo3D. They merely construct a body-fitting human model through parametric settings of human characteristics to achieve the reverse transformation of the pattern from 3D to 2D. The digital import of real knitted fabrics is not addressed, and the fabric properties still rely on the software’s default settings [5].

Although there is an issue of insufficient synergy in the digital fields of sports underwear and knitted fabrics, some scholars are exploring solutions. A practical survey by scholars such as Zhang Hui among underwear enterprises shows that insufficient digital precision of fabrics and incompatibility between scanning data and simulation software” are the two primary factors restricting the implementation of technology. Over 60% of enterprises still rely on experience for parameter adjustment, highlighting the industrial demand for collaborative simulation technology of fabrics and clothing [16]. Zhan Biqin et al. conducted research on the component-based virtual display of fully-fashioned dual layer knitted clothing, and established the topological mapping relationship between two-dimensional patterns and three-dimensional models [19]. Notably, Turkish scholars Ermin and Şen Kılıç approached from the perspective of functional structure. Through supportive designs such as wide shoulder straps, high side wings, and inner lining pockets, they constructed a dynamic stability system for breast-form bras in Clo3D. They also verified the fit performance of these bras under virtual motion postures by combining pressure maps [20].

This study aims to address bottleneck issues in the current digital development of sports underwear, such as fabric simulation distortion, disconnection between dynamic and static designs, and parameter reliance on experience. To this end, this study focuses on a cotton/spandex (78%/22%) rib-knitted fabric commonly used in sports bras and proposes to construct a full process digital simulation method for knitted sports underwear based on the collaboration of TG3D-Nuno and CLO3D. (In this study, we used CLO3D Version 7.0, developed by CLO Virtual Fashion Inc., Seoul, South Korea.) Specific objectives include: achieving high-fidelity digital restoration of rib-knitted fabrics; establishing a mapping and calibration mechanism from scanning data to simulation parameters; developing a functional sports underwear pattern suitable for running scenarios and verifying its fit and support performance.

## 2. Experiment preparation

### 2.1. Experimental workflow, materials and equipment

**Fig 1:**
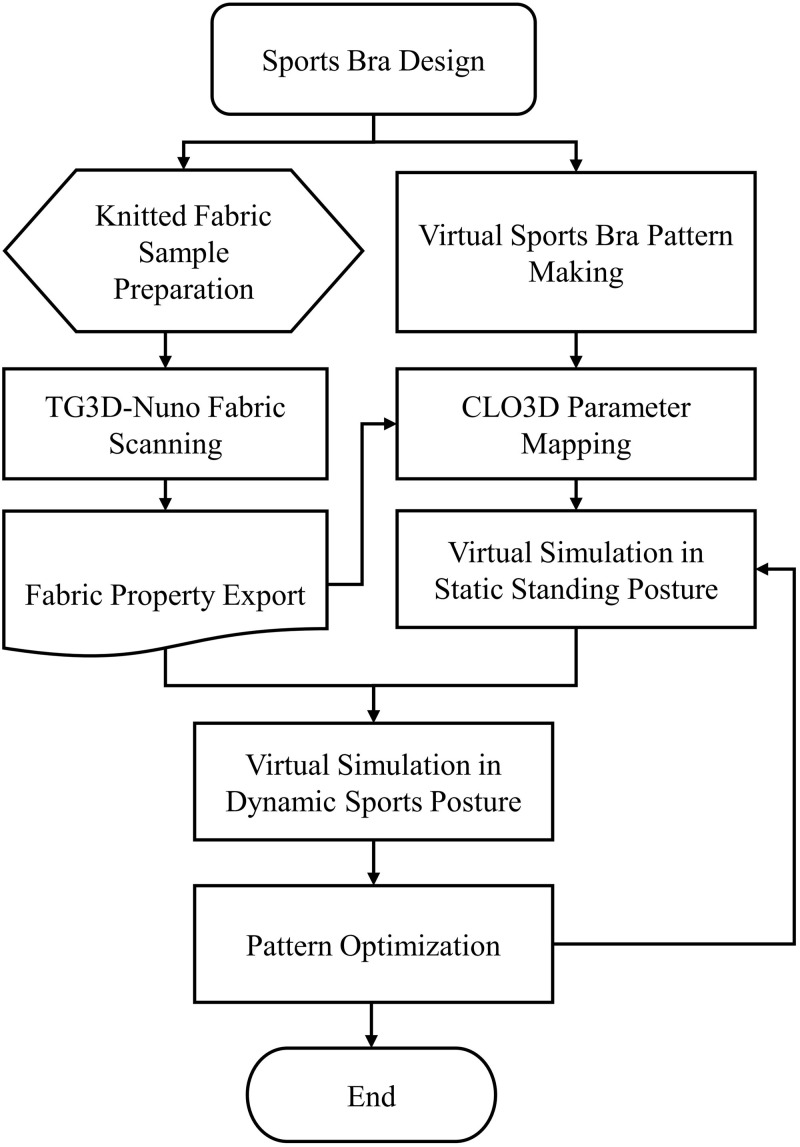
Schematic diagram of the research workflow for virtual sports bra design.

(1) Experimental Workflows:

In this study, we established an integrated workflow for sports bra development, as visually summarized in Fig 1. The workflow synergistically combines two parallel streams: (i) the digitization and physical calibration of knitted fabric via TG3D-Nuno scanning and CLO3D parameter mapping, and (ii) the virtual design and static fitting of the bra pattern. These streams converge into a core validation and optimization loop, where the digitally assembled prototype undergoes dynamic simulation in a sports posture, leading to targeted pattern refinement. This process forms a closed-loop from physical material to a functionally validated virtual prototype.

**Fig 2:**
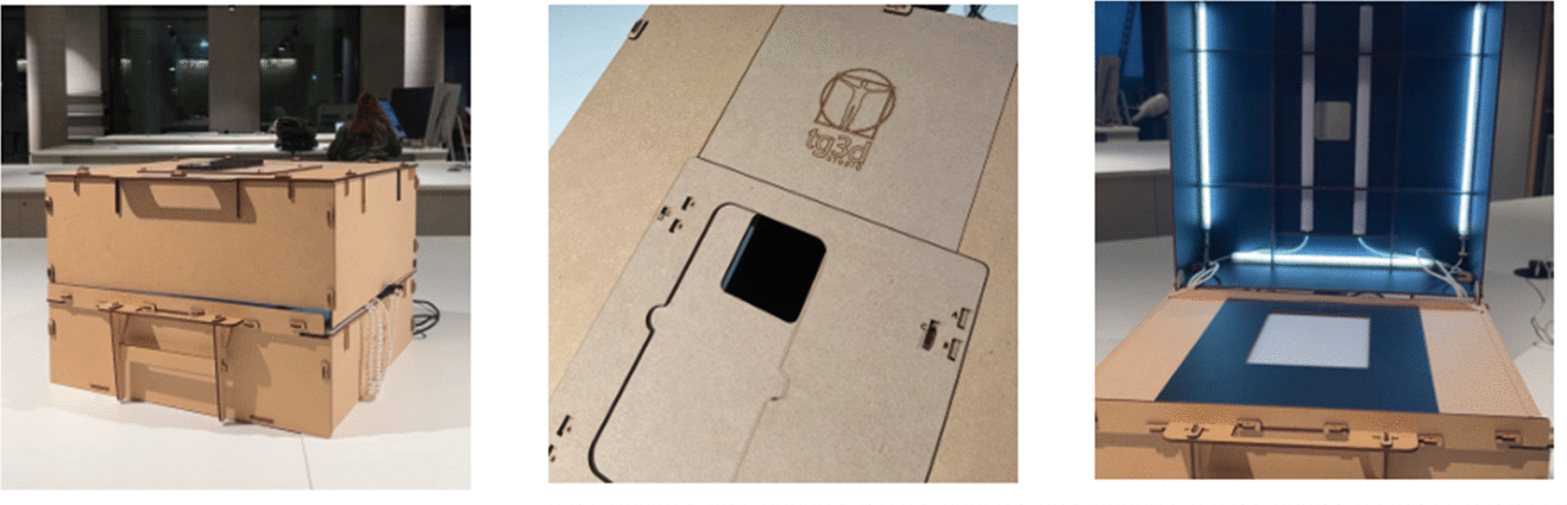
TG3D Scantic nuno fabric scanning box.

(2) Experimental Materials: The mainstream rib knitted fabric in the sports bra market was selected as the research object. This fabric is composed of 78% cotton and 22% spandex, and possesses excellent elastic recovery property and support performance, which meets the functional requirements of sports bras.
(3) Fabric Scanning Equipment: The TG3D ScanticNuno Fabric Scanning Box as shown in Fig 2, with the supporting scanning terminal being an iPhone 13 (pre-installed with the *NunoScan* APP).

### 2.2. TG3D-nuno scanning process and parameter settings

**Fig 3:**
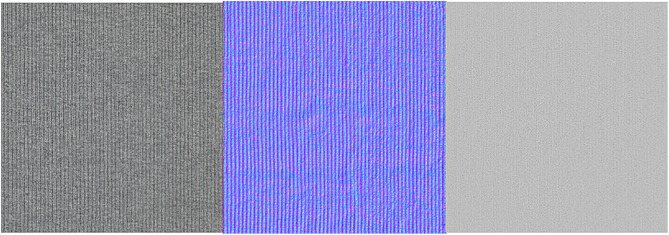
Texture Maps of Knitted Fabrics. (From left to right are the Base Color Map, Normal Map, and Roughness Map respectively).

The experiment utilized the TG3DScanticNuno scanning system to accomplish the digital collection of fabrics. This system realizes the 3D texture restoration of fabrics through multi-angle light source projection and image synthesis technology. The specific operation process strictly adheres to the equipment technical specifications, and the specific steps are as follows:

1. Preparation of the scanning environment: Place the TG3D scanning box on a horizontal workbench. Lay the pre-balanced knitted fabric sample flat in the center of the acrylic platform at the lower layer of the scanning box. Fix the four corners of the fabric with transparent pressing plates to ensure that the fabric has no wrinkles, the yarns are not displaced, and the edges of the fabric are aligned with the scale lines of the platform, which is convenient for subsequent size calibration.
2. Setting and Execution of Scanning Parameters: In this step, open the NunoScan APP on an Apple iPhone 13 and establish a connection with the scanning box via Bluetooth. Then enter the parameter-setting interface. Subsequently, close the door of the scanning box to avoid interference from external light. Click the “Start Scanning” button on the APP, and the device will automatically complete the image collection and synthesis from 12 angles. The entire scanning process takes approximately 90 seconds.
3. Digital Asset Export and Pre-processing: Log in to the official TG3D cloud platform and enter the fabric editing interface. Select the target fabric area with a marquee, check the core attribute files such as “Color Map, Normal Map, Roughness Map, Displacement Map”, and click “Batch Export” to obtain the digital asset package. Use the “Cropping Tool” to remove the invalid edge areas to ensure compatibility with the material import requirements of Clo3D. Some of the pre-processed core map files are shown in Fig 3. The base color map clearly presents the color hierarchy of the ribbed texture. The normal map simulates the bumpy texture of the fabric surface through gray-scale differences, and the roughness map reflects the reflection differences in different areas of the fabric.

### 2.3. Evaluation of digital restoration effect

**Fig 4:**
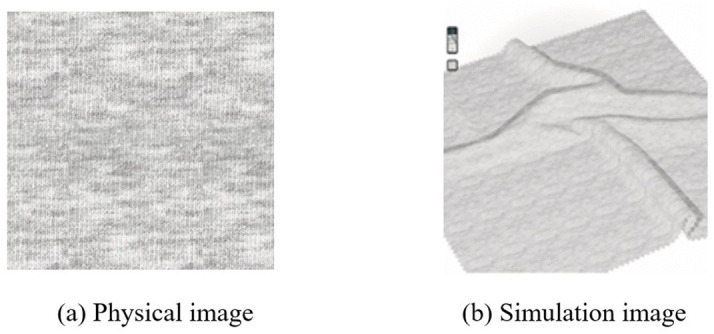
Physical image (a) and simulation image (b) of ribbed knitted fabric.

Verified by physical comparison and virtual simulation results, the digital fabrics obtained using the TG3D-Nuno scanning system can highly reproduce the appearance of physical fabrics. As shown in the physical and simulation images in Fig 4, the simulation effect visually exhibits good consistency with the actual fabric. In terms of texture details, the shape of the ribbed loops and the yarn direction of the virtual fabric closely match those of the physical fabric, with no obvious deformation or distortion. Regarding color and texture, the matte property of the virtual fabric is consistent with the tactile and visual feedback of the physical fabric. The color of the fabric in the simulation image was modified twice to better meet the design requirements. After applying this virtual fabric to the sports underwear pattern, the transition of the fabric texture at the curved surface of the garment is natural, and the arrangement direction of the ribbed loops is adapted to the garment structure. There is no texture distortion caused by stretching deformation, demonstrating that the technical path of combining the TG3D-Nuno scanning system with Clo3D parameter adjustment can achieve high-fidelity restoration of ribbed knitted fabrics from physical to virtual, providing reliable digital support for the realistic design of sports underwear.

## 3. Construction of sports underwear pattern

The virtual design and pattern construction experiment of sports underwear was carried out based on the results of the knitted fabric simulation experiment and the Clo3D software. Thanks to its mature virtual human body modeling and dynamic physics engine, the Clo3D software has become a mainstream platform in digital fashion design [5,20,21]. As a mainstream tool in current digital fashion design, it features a mature physical simulation engine, capable of fairly realistically reproducing the interactive behavior between fabrics and the human body in dynamic states. The process of this experiment successively encompasses four steps: material parameter mapping and calibration, pattern construction, and motion form simulation.

### 3.1. Parameter mapping and calibration of knitted fabrics based on CLO3D

Various texture maps collected and pre-processed by the TG3D-Nuno system, including color maps, normal maps, roughness maps and displacement maps, were imported into the Material Editor of CLO3D in accordance with corresponding attribute classifications. Through parameter mapping and global data calibration, the original fabric scanning data was converted into a standardized parameter system compatible with the CLO3D simulation environment. During the whole parameter configuration process, all attribute items were inspected and corrected item by item to ensure the rationality and accuracy of material settings. The standardized parameters adapted to the CLO3D simulation environment are summarized in Table 1, which covers the basic information, material properties, texture mapping settings, optical indexes, physical performances and structural characteristics of rib-knitted fabrics, including fabric name and weaving type, matte material attribute, intensity parameters of normal map and displacement map, particle distance, opacity, roughness and reflection intensity, metalness, fabric thickness, as well as texture width, height and rotation angle.

**Table 1:** Calibrated CLO3D material parameters of rib-knitted fabric based on TG3D-nuno scanning.

Classification	Parameter Item	Value/Setting
Basic Information	Name	Rib_2X2_468g
	Construction	Knit
Material	Type	Fabric_Matte
Normal Map	Intensity	10
Displacement Map	Amount	3.00
	Particle Distance	4.00
Opacity	Opacity Value	100
Roughness	Map Intensity	54
	Reflection Intensity	15
Physical Properties	Metalness	0
Texture Transformation	Thickness	1.49mm
Basic Information	Width	113.2
	Height	117.4
	Angle	0.00

After completing the above basic parameter configuration and systematic verification, it provides solid and standardized data support for subsequent virtual pattern design and dynamic simulation analysis of garments.

### 3.2. Design and pattern construction of digital sports underwear in a standing posture

Open a female virtual human model in Clo3D. The anthropometric measurements used for the virtual model were based on the standard female body size provided in the CLO3D avatar library as shown in Table 2.

**Table 2:** Dimensions of main parts of the virtual female model (cm).

Measurement Items	Value	Measurement Items	Value	Measurement Items	Value
Total height	175.3	CF neck to waist	34.0	Inseam length	84.1
Chest circumference	80.6	CB neck to waist	38.1	Thigh circumference	54.9
Distance from nipple to HPS	25.1	High hip circumference	80.3	Shoulder-neck to wrist	77.8
Base neck circumference	35.6	Low hip circumference	95.6	Upper arm circumference	25.4
Shoulder width	37.8	Total crotch depth	72.4	Under bust circumference	66.2

Sports bras are generally classified into compression-style and encapsulation-style designs. Compression-style sports bras stabilize the breasts by pressing them against the chest wall, thereby reducing excessive displacement during physical activity. In contrast, encapsulation-style sports bras provide support by individually supporting each breast and distributing the load through structured components such as straps and panels [22]. Considering the advantages of both approaches, this study integrates the design principles of compression-type and encapsulation-type bras in order to develop a sports bra that provides improved stability and comfort during exercise.

**Fig 5:**
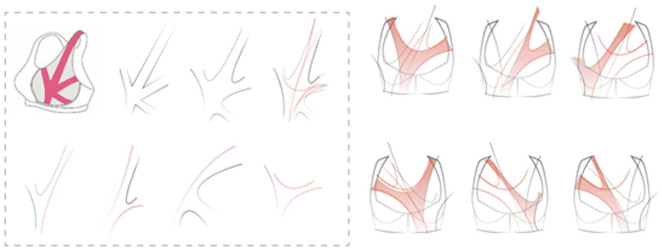
Extraction of triangular support paths from the CW-X sports bra structure.

The structural concept of the proposed design was inspired by the internal support configuration of the CW-X sports bra. As illustrated in Fig 5, key force-transmission paths were extracted from the internal support structure and simplified into diagonal support lines. These lines form triangular force-bearing pathways across the chest, which informed the development of the triangular panel system adopted in this research. Compared with conventional two-strap structures that primarily rely on vertical shoulder tension, the triangular configuration distributes forces along multiple directions and increases the effective load-bearing area, thereby improving overall structural stability.

**Fig 6:**
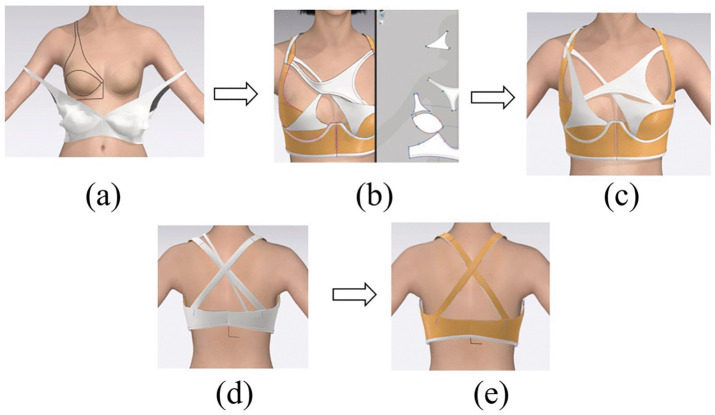
Pattern development and structural optimization process of the sports bra using the CLO3D “3DPen” tool. (a) Initial wire-free bra outline; (b) introduction of triangular front panels; (c) repositioning of panel endpoints to the lower lateral breast; (d) addition of crossed back straps; (e) formation of a front–back linked support system.

Key force-transmission lines were identified from the internal five-point support configuration and simplified into diagonal support paths, which informed the design of the triangular panel system. Based on the extracted support paths, the initial structural concept was further developed through digital pattern construction using the “3DPen” tool in CLO3D, which is commonly used for creating free-form garment structures in virtual prototyping [23]. The “3DPen” tool allows designers to directly sketch structural lines on the digital body surface and convert them into garment panels. In this study, the tool was used to generate the initial pattern lines and construct the panel configuration of the sports bra. The pattern development and iterative adjustment process are shown in Fig 6.

In the first stage (Fig 6a), the outline of a wire-free basic bra was drafted to form a preliminary wrapping structure around the chest. However, due to insufficient structural support and fabric elasticity, the cup shifted downward when tested on the model, indicating that the basic structure could not effectively stabilize the breast during movement.

To address this issue, a triangular panel structure was introduced across the shoulders and the front chest in the second stage (Fig 6b). The purpose of this configuration was to increase the load-bearing area and guide the support force along diagonal directions derived from the extracted support paths. Nevertheless, the initial triangular panel extended to the lower edge of the bra and failed to adequately support the lower portion of the breast. As a result, the force transmission remained inefficient and the lifting effect was limited.

Therefore, in the third stage (Fig 6c), the endpoints of the triangular panel were repositioned to the lower lateral region of the breast. This modification established a clearer bottom-to-top support pathway, enabling the triangular structure to generate lifting forces from beneath the breast and significantly improving the support performance.

Subsequently, a crossed-strap configuration was introduced on the back (Fig 6d). By overlapping the shoulder straps above the scapular region, the overall wrapping effect and anti-displacement stability of the garment were enhanced. This configuration allowed the front and back structures to function as an integrated support system (Fig 6e) [21].

During the optimization process, it was also observed that triangular panels without reinforcement were prone to deformation under stress, which resulted in irregular stress distribution in the fabric and affected the overall fit. After structural adjustments, the front chest panels achieved a more stable and natural fit. However, the support generated at the front could not effectively extend to the back, and the mechanical load was still primarily borne by the shoulder straps.

To improve the continuity of the support system, the triangular panels on the front chest were further extended toward the back, forming a circular force-bearing structure across the upper torso. This configuration allows the forces generated at the front to be transmitted to the back, thereby improving the overall stability of the garment. In addition, an extra shoulder strap was introduced to enlarge the contact area on the shoulders. According to the principle of pressure distribution, increasing the contact area can reduce localized pressure and enhance wearing comfort.

A vertical zipper was also installed at the center of the front chest, extending from the hem to below the collar, allowing users to easily put on and remove the garment. The edge of the cup smoothly follows the contour of the chest, avoiding excessive compression. Above the cup body, a set of diagonal crossed straps extends from both sides of the chest and converges at the back of the neck, further enhancing lifting force and structural stability.

**Fig 7:**
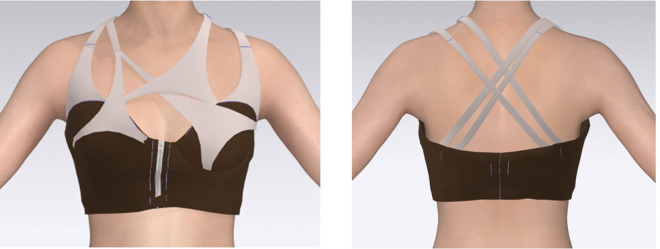
Virtual Simulation Effect of Sports Underwear in Standing Posture.

Finally, the virtual simulation result of the sports bra in a standing posture is presented in Fig 7, demonstrating the overall structural configuration and fitting performance of the proposed design.

## 4. Optimization of sports underwear pattern

If a sports bra does not properly conform to the human body, its functional performance and wearing comfort may be significantly affected [24]. To improve the consistency between digital simulation and actual wearing scenarios, this study conducted pattern optimization through an iterative process combining motion-driven simulation and pattern refinement.

**Fig 8:**
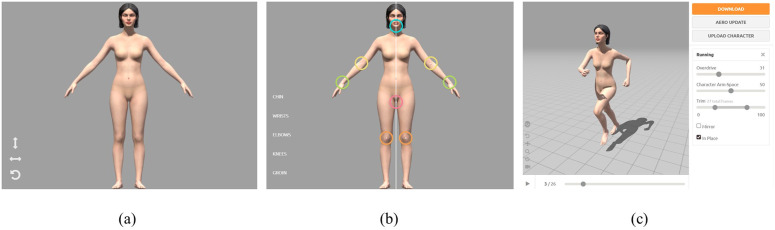
Workflow of motion-driven garment simulation. (a) CLO3D avatar exported for animation; (b) skeletal landmark mapping for animation rigging; (c) generation of running animation in *Mixamo*.

To generate a realistic dynamic motion, the virtual human model created in CLO3D was exported and imported into the *Mixamo* animation platform. By mapping key skeletal landmarks of the avatar, a predefined female running animation was applied to the model. The animated avatar was then imported back into CLO3D to drive the garment simulation. The workflow of the animation-driven simulation process is illustrated in **Fig 8**.

**Fig 9:**
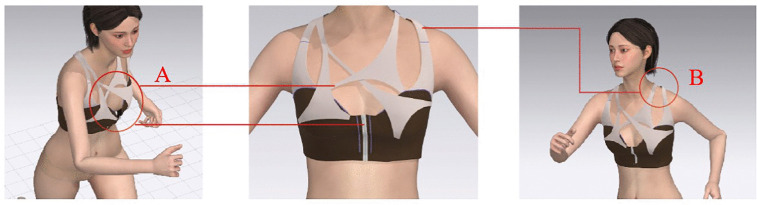
Structural problems identified in the initial sports bra design during running simulation. (A) asymmetric shoulder strap causing insufficient lifting force and cup gaping; (B) shoulder strap loosening and slipping during motion. The middle image presents a magnified view of the chest area highlighting garment deformation.

During the dynamic simulation stage, particular attention was paid to the **stress–deformation behavior of the cross-panel structure on the front chest**, the **dynamic stability of the elastic ribbed hem**, and the **interaction between the shoulder straps and the cups** [25]. The running simulation revealed several structural problems in the initial design, as shown in **Fig 9 below**.

First, as indicated in **Fig 9 A part**, the asymmetric configuration of the left shoulder strap resulted in an imbalance in the distribution of support forces between the two sides. This imbalance reduced the lifting force of the cup and produced a gap between the cup and the breast. Second, as illustrated in **Fig 9 B part**, the left shoulder strap tended to loosen and slip during running, indicating that the original strap configuration could not maintain stable tension during dynamic movement.

These issues were associated with the structural characteristics of the initial pattern design. Although the design concept was derived from the triangular support paths extracted from the CW-X sports bra structure (as discussed in the previous section), the early pattern still required further refinement to achieve balanced force transmission and stable support during motion.

**Fig 10:**
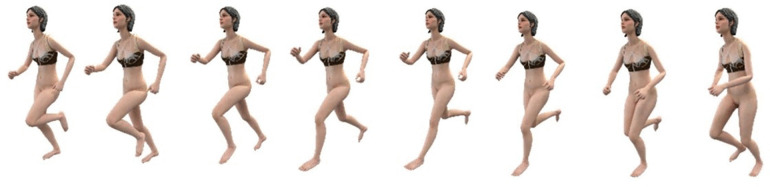
Representative key frames of the running motion sequence used for dynamic garment simulation.

To address these issues, the pattern was optimized by adjusting the symmetry of the shoulder straps and refining the contour lines of the cup and side panels. After several rounds of pattern modification and simulation verification, the optimized sports bra demonstrated improved structural stability during running motion. The running motion sequence used for the dynamic simulation is illustrated in **Fig 10**.

The animation-driven motion was applied to evaluate garment stability and deformation during dynamic movement.

**Fig 11:**
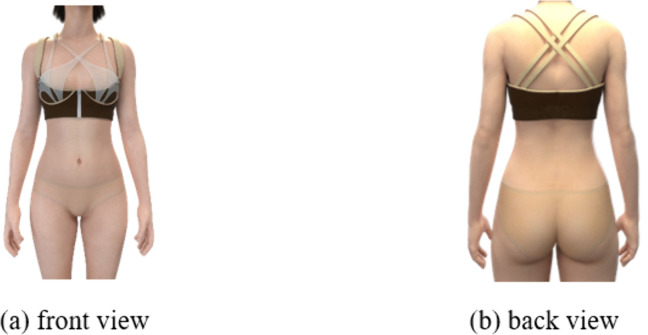
Virtual simulation results of the optimized sports bra design under a standing posture.

Furthermore, the optimized design was also evaluated under a neutral standing posture to verify its fit in static conditions. As shown in Fig 11, the shoulder straps exhibit balanced force distribution, and the cups closely conform to the curvature of the chest. No significant wrinkling accumulation was observed in the armpit or side areas, indicating that the optimized pattern provides improved fit and structural stability. Although the simulation is based on animation-driven motion rather than full biomechanical modeling, it provides a practical and effective method for evaluating garment behavior during dynamic movement. The results demonstrate improved fit and balanced support distribution after pattern optimization.

### 4.1. Comparison of pattern pieces of sports underwear before and after optimization

**Fig 12:**
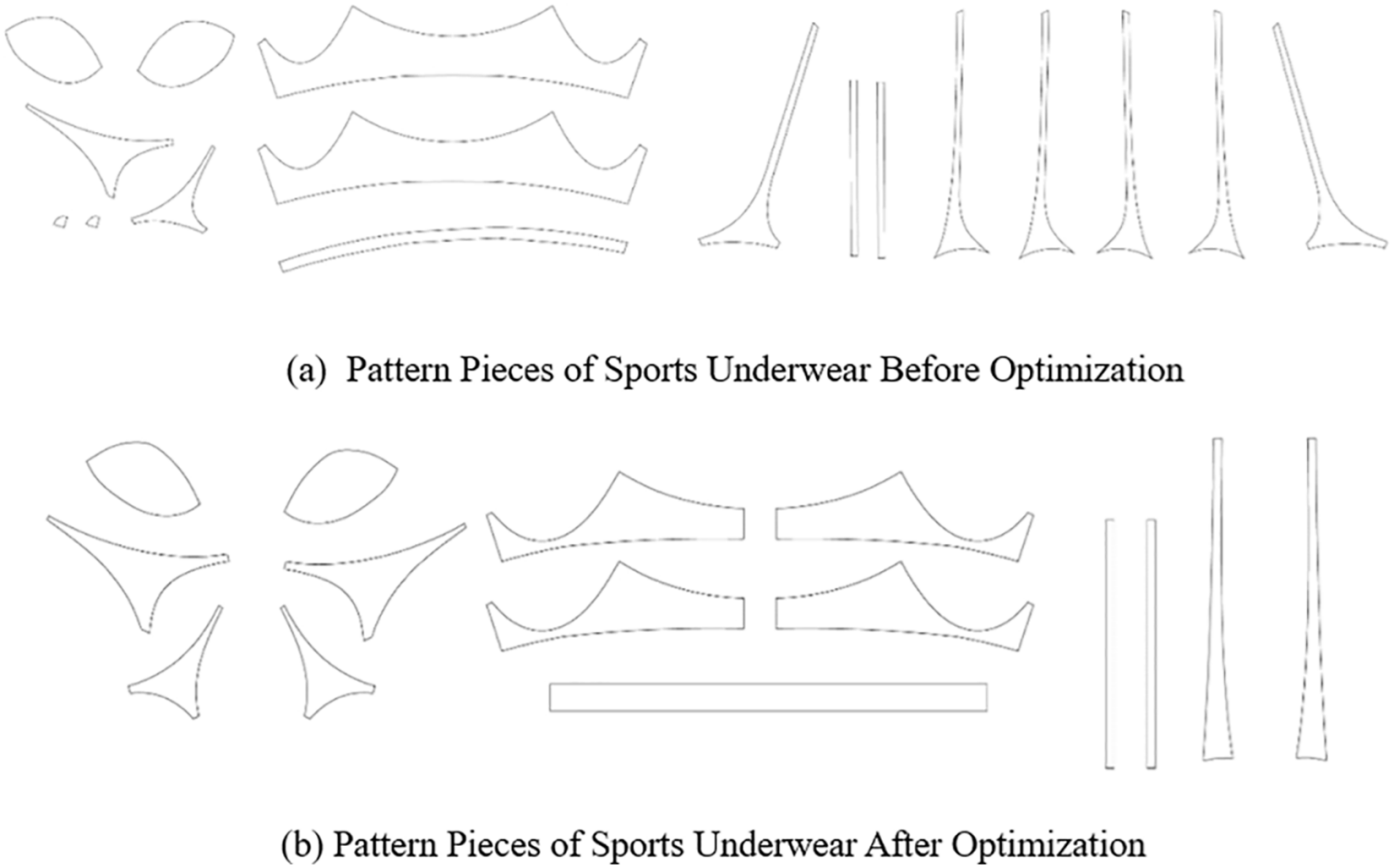
Comparison of Sports Underwear Pattern Pieces Before and After Optimization.

As shown in Fig 12(a), before optimization, the pattern outline of the sports underwear was relatively complex, with redundant cut-pieces and unconventional splicing lines in some areas. Especially in the connection area between the shoulder straps and the front chest, as well as at the transition of the hem, the pattern segmentation was rather complicated, and the seam directions did not fully conform to the main curvatures and movement-stretching directions of the human body. Such structures not only increase the difficulty of the sewing process but may also cause local deformation or discomfort due to stress concentration during dynamic wearing.

In contrast, the optimized pattern presented in Fig 12(b) significantly trends towards simplicity and refinement in form. Its main characteristics are reflected in the rational integration of structural lines. Redundant cut-pieces are combined, and the contour lines of key functional areas are smoother and more fluent. From the perspectives of design logic and manufacturing feasibility, the simplicity of the optimized pattern not only enhances the visual sense of order but also brings multiple benefits in practical applications. On the one hand, fewer cut-pieces and a more reasonable splicing method help simplify the production process and improve fabric utilization. On the other hand, the streamlining and optimization of structural lines reduce the potential accumulation of sewing errors, contributing to ensuring the consistency of finished products. More importantly, the refinement of the pattern does not come at the expense of functionality. Instead, through the fine calibration of key curves and the pre-control of fabric stretching directions, the optimized pattern achieves better fit, support, and motion adaptability in the three-dimensional wearing state.

### 4.2. Parametric comparison of the patterns of sports underwear before and after optimization

Quantitative evaluation of critical dimensional parameters is essential to verify the rationality of structural adjustment and pattern optimization for functional sports bras. To objectively quantify the fitting performance between garment patterns and human body dimensions, this study proposes the Dimensional Matching Index (DMI) as a quantitative evaluation indicator. The DMI can intuitively reflect the dimensional matching degree of key clothing areas by normalizing the deviation between pattern size and human body reference size. The specific calculation formula is as follows:


$$\begin{array}{c} \text{DMI}=\left(\text{1}-\frac{\left|L_{\text{pattern}}-L_{\text{body}}\right|}{L_{\text{body}}}\right)\times \text{1}\text{0}\text{0}\% \end{array}$$ (1)

In Formula (1), L_pattern_ refers to the measured size of the pattern piece in the 2D flat state or the 3D virtual wearing state; L_body_ represents the standard benchmark size of the corresponding body area of the virtual female model. The DMI value ranges from 0% to 100%, and a higher value indicates a smaller dimensional deviation and better contour fitting performance between the sports bra and the human body.

Combined with the functional characteristics of running sports bras, four core adaptive areas closely related to wearing comfort and support stability were selected for comparative analysis, including under-bust circumference, chest cup bottom fitting line, cup contour fitting line and single-side crossed shoulder strap fitting line. The 2D plane development size and 3D dynamic wearing size of the original pattern and the optimized pattern were compared respectively, and all dimensional benchmark data of the virtual model were unified as the measurement standard. The detailed original dimensional comparison data of each functional area are summarized in Table 3.

**Table 3:** Comparison of parameters of key dimensions before and after optimization.

Adaptation Area	Reference Item	Model Benchmark (mm)	Pattern Size before Optimization (mm)	Pattern Size after Optimization (mm)
Under-bust circumference	2D	662.7	644.5	609.2
	3D	662.7	666.4	653.2
Chest Cup Bottom Fitting Line Cup Contour Fitting Line	2D	482	466.2	485.8
	3D	482	470.7	483.6
Single-side Crossed Shoulder Strap Fitting Line Chest Cup Bottom Fitting Line Cup Contour Fitting Line	2D	219.65	228.2	206.1
	3D	219.65	214.3	229.7
Single-side Crossed Shoulder Strap Fitting Line	2D	485.9	466.7	460.9
	3D	485.9	494.7	509.6

Based on the original size data in Table 3 and combined with the DMI calculation formula, the dimensional matching index of each key functional area before and after pattern optimization was calculated uniformly. The statistical results of DMI values are shown in Table 4, which visually presents the improvement of fitting accuracy after structural iteration and parameter adjustment.

**Table 4:** Dimensional matching index of key functional areas before and after optimization.

Adaptation Area	DMI before Optimization (%)	DMI after Optimization (%)
Under-bust circumference (3D)	99.4	98.6
Chest Cup Bottom Fitting Line (3D)	97.7	99.7
Cup Contour Fitting Line (3D)	98.5	98.7
Single-side Crossed Shoulder Strap Fitting Line (3D)	98.2	95.5

It can be clearly seen from the data in Table 3 and Table 4 that the overall dimensional matching performance of the optimized pattern has been significantly improved in core support areas. For the chest cup bottom fitting line, which undertakes the main lifting and supporting function, the DMI value increased from 97.7% to 99.7% after optimization, realizing nearly precise dimensional matching with the human body contour, and effectively solving the problems of local gaping and insufficient wrapping of the cup in the initial design. The DMI of the cup contour fitting line also increased slightly from 98.5% to 98.7%, proving that the optimized cup structure can better fit the breast curvature and improve the comprehensive wrapping performance.

The 3D matching degree of the under-bust circumference remained above 98% before and after optimization. The optimized 2D flat size was appropriately reduced to reserve reasonable elastic shrinkage for the rib-knitted fabric. This active control of pattern allowance makes the fabric produce stable and uniform tensile stress in the 3D wearing state, balancing close-fitting effect and long-term wearing comfort. The DMI of the crossed shoulder strap decreased slightly to 95.5%, which is a reasonable design trade-off. Appropriately expanding the 3D size of the shoulder strap can provide reserved stretching space for limb movement during running, avoiding tight constraint and strap breakage while ensuring basic anti-slip performance.

In addition, there are obvious systematic differences between 2D flat size and 3D virtual wearing size. This difference is mainly caused by the elastic deformation of knitted fabrics, the bending and stretching of garment panels along the human body’s curved surface, and the physical engine stress calculation in CLO3D. The 2D size focuses on the structural rationality of pattern cutting, while the 3D size truly reflects the final morphological state of the sports bra in actual wearing scenarios. After unified optimization, the coordination of 2D design and 3D fitting of all functional areas has been greatly enhanced.

In summary, the collaborative analysis of original dimension parameters and DMI quantitative indicators verifies the effectiveness of the pattern optimization strategy. The revised structural lines and dimensional parameters significantly optimize the dynamic fitting, force transmission efficiency and movement adaptability of the sports bra, which provides reliable quantitative support for the digital precise design of knitted functional underwear.

## 5. Conclusion

This study established a full-process digital workflow for knitted sports bras by combining TG3D-Nuno fabric scanning and CLO3D dynamic simulation. Results confirmed that high-fidelity virtual reproduction of rib-knitted fabrics can be achieved through scanning and parameter calibration. Using a standard female virtual human model, the workflow realized 3D pattern design, static fitting, and dynamic verification under running motion, enabling intuitive identification and correction of defects such as strap slippage and insufficient cup fit. The Dimensional Matching Index (DMI) was introduced to quantitatively evaluate pattern–body matching, with key areas exceeding 98% after optimization.

Compared with the traditional garment development model and process, which relies on manual sampling, repeated physical cutting, and real fabric testing, the proposed digital method helps reduce potential costs, labor, and time during the early design stage. It supports efficient design iteration and early functional verification, providing a practical technical route for the rapid, precise, and sustainable development of functional sports underwear. Potential extensions to specialized intimate apparel may be explored with further validation.

### 5.1. Limitations

Several limitations should be noted. First, the fabric model mainly reproduces visual appearance and basic deformation, while dynamic pressure distribution and complex mechanical responses under real wearing conditions are not fully captured. Second, conventional virtual simulation software cannot completely represent refined sewing details, including seam allowances, sewing threads, and professional underwear manufacturing techniques such as coverstitching and overlocking. Third, dynamic verification was limited to running motion, and other high-intensity sports scenarios were not included. These constraints reflect common technical bottlenecks in virtual garment simulation and do not affect the validity of the proposed workflow.

### 5.2. Future work

Future research will focus on improving the comprehensiveness and realism of the proposed framework. Dynamic pressure evaluation will be incorporated to establish a complete evaluation system that considers both support performance and wearing comfort. The representation of complex sewing structures, seam details, and yarn mechanical properties will be refined to further enhance the fidelity of virtual simulation. The workflow will be extended to cover a wider range of motion scenarios, including jumping, rapid direction changes, and limb stretching, and will be adapted to diverse body shapes to strengthen its industrial applicability for general functional sports bras. In addition, the digital workflow will be further integrated with physical sample production to narrow the gap between virtual simulation and real garment performance. On this basis, the optimized technical framework can provide a feasible technical reference for the subsequent design and performance optimization of specialized functional undergarments. Relevant exploration targeting post-operative rehabilitation bras will be carried out in follow-up independent studies with targeted experimental verification, so as to avoid over-generalization beyond the current research scope.
